# Where Wnts Went: The Exploding Field of Lrp5 and Lrp6 Signaling in Bone

**DOI:** 10.1359/jbmr.081235

**Published:** 2008-12-15

**Authors:** Bart O Williams, Karl L Insogna

**Affiliations:** 1 Van Andel Research Institute Grand Rapids, Michigan, USA; 2 Department of Internal Medicine, Yale University School of Medicine New Haven, Connecticut, USA

**Keywords:** Lrp5, Lrp6, Wnt, β-catenin, osteoporosis

## Abstract

Wnt signaling has emerged as a central regulator of skeletal modeling and remodeling. Loss- or gain-of-function mutations in two Wnt co-receptors, Lrp5 and (more recently) Lrp6, have drawn attention to the importance of the Wnt pathway in bone biology. This review summarizes our current understanding of how the Wnt pathway operates on bone and the implications this has for skeletal physiology and drug discovery. Over the past 9 yr, rapid advances have been made in our understanding of the cellular targets for Wnt signaling and of the important regulatory molecules in this metabolic pathway. Both canonical and noncanonical signaling pathways seem to be important for mediating the effects of Wnt in bone. A rapidly expanding catalog of genetically engineered mice has been used to establish the importance of downstream effector molecules (such as β-catenin) in the Wnt pathway, as well as the critical role of endogenous inhibitors of Wnt signaling (such as Dkk1 and sclerostin) in bone metabolism. Indeed, regulation of sclerostin in osteocytes is emerging as an important final pathway for regulating bone anabolism in response to diverse trophic stimuli, from mechnotransduction to the anabolic actions of PTH. From the outset, it had been assumed that the effects of Wnt signaling in bone were caused by direct actions in osteoblast precursors, osteoblasts, and osteocytes. However, startling recent findings have challenged this view and suggest that a key target, at least in mice, is the duodenal enterochromaffin cell. There, Wnt signaling transduced by Lrp5 regulates serotonin synthesis, which acts in an endocrine fashion to regulate bone cell metabolism. It will take time to reconcile this new information with the considerable body of information we already have regarding the actions of Wnt in bone. The Wnt pathway has rapidly emerged as a therapeutic target for drug discovery. Neutralizing antibodies and small-molecule inhibitors of endogenous Wnt inhibitors have shown early promise as bone anabolic agents. However, given the central role of the Wnt pathway in regulating growth and development in extraskeletal tissues, as well as our still rudimentary understanding of how this signaling cascade actually affects bone metabolism, considerable work will be needed to ensure the safety of these new therapies.

## INTRODUCTION

When the low-density lipoprotein-related receptor 5 and 6 (*Lrp5* and *Lrp6*) genes were cloned in 1998 based on their homology with the low-density lipoprotein receptor (LDLR),(1–4) it would have been difficult to anticipate the key role that they play in skeletal development and disease. Mutations in either *LRP5* or *LRP6*, proteins with which they directly interact, or their downstream signaling targets have been associated with several bone-related diseases, as well as with many types of cancer,(5) abnormalities of the eye,(6) neurodegenerative disease,(7) congenital heart defects,(8) and metabolic disorders.(9) The focus of this perspective will be to briefly describe the structure and functions of Lrp5/Lrp6 and their role in skeletal development and remodeling, followed by a summary of areas of current research and future investigative efforts in this exciting new area in bone biology.

## STRUCTURE OF Lrp5 AND Lrp6

Lrp5 and Lrp6 are 71% homologous and form a subclass of the LDLR family.(10) They share a structure that contains a large extracellular domain (ECD) making up >85% of the ∼1600-amino-acid proteins. At the amino terminus of the ECD, four β-propeller motifs alternate with four epidermal growth factor (EGF)-like repeats to create binding sites for most of the known extracellular ligands for Lrp5 and Lrp6. Structural models suggest that each of these β-propeller domains resembles the YWTD/EFG domain structure of the LDLR, which has a six-bladed propeller structure where each blade consists of ∼43 amino acids. In a recent model of the Lrp5 structure, Bhat et al.(11) described the β-propeller as “a disk with inward-sloping sides surrounding a central open area.” Another way to visualize this would be to consider this as a funnel-like structure.

Following the four β-propeller motifs are three repeats of ∼38 amino acids highly homologous to lipoprotein ligand-binding domains in the LDLR. The precise function of these repeats is not entirely understood; however, there is much circumstantial evidence to support an important physiological role. For example, there is evidence that Wnt ligands are presented to these receptors in the form of a lipid-rich complex.(12) In addition, Lrp5 has been shown to mediate the binding of apolipoprotein particles to cells,(4) and both *Lrp5*-deficient mice(13,14) and humans with mutations in LRP6(15) have elevated levels of plasma LDL and triglycerides, suggesting that these molecules may play direct roles in regulating lipid metabolism.

The ECD is anchored to the plasma membrane through a 22-amino-acid single-pass transmembrane domain, which is followed by a relatively short (207 amino acids) cytoplasmic domain. The most obvious motifs in the cytoplasmic domain are a series of highly conserved PPPS/TP motifs. The serine or threonine residues in these proline-rich regions are phosphorylated on activation by Wnts or other ligands such as PTH (see below). These phospho-serine or phospho-threonine residues serve as binding sites for the axin proteins, resulting in stabilization of β-catenin.(10) In addition, Wnt-induced activation of Lrp5 or Lrp6 can directly activate the mammalian target of rapamycin (mTOR) pathway,(16) raising the possibility that some phenotypes associated with activation of this pathway could be ameliorated through treatment with rapamycin.

The expression of an alternatively spliced form of *LRP5* may also play a role in regulating the actions of this co-receptor. One of the first reports on Lrp5 (originally referred to as *Lrp7/Lr3*) described an alternatively sized mRNA expressed during embryogenesis and in the testis.(17) More recently, Pospisil et al.(18) also reported evidence for splicing of Lrp5. The alternatively spliced form of Lrp5 directs the synthesis of a protein that lacks the third β-propeller domain and that is not subject to regulation by the Dkk1 protein. An alternatively spliced form of Lrp5 has also been identified in parathyroid tumors.(19) Given the structural complexity of the Lrp5 protein, it is tempting to speculate that these alternative transcripts have important roles during development and perhaps in the pathogenesis of some neoplasms, possibilities that merit more study.

## REGULATION OF Lrp5 AND Lrp6 ACTIVITY

The most studied functions of Lrp5 and Lrp6 are their roles as co-receptors for the frizzled family of Wnt receptors. Their Wnt ligands are a family of proteins encoded by 19 different genes in vertebrates. The Wnt signaling pathway is one of the most highly conserved signaling cascades identified with functional activity in organisms including, but not limited to, Hydra, planaria, *Drosophila*, *Caenorhabditis elegans*, and all vertebrate species examined.(20)

Wnts are a family of cysteine-rich secreted glycoproteins(20) that induce signals through several intracellular cascades. In the most studied (“canonical”) pathway, Wnt binds to a receptor complex that includes both a member of the frizzled family of seven-transmembrane receptors (encoded by 10 genes in humans) and either Lrp5 or Lrp6.(10) This results in phosphorylation of the intracellular cytoplasmic tail of Lrp5 or Lrp6, creating a binding site for the Axin protein. Normally, Axin is part of an intracellular protein complex that facilitates the phosphorylation of β-catenin by glycogen synthase kinase 3 (GSK3), thereby targeting β-catenin for ubiquitin-dependent proteolytic degradation. In the presence of Wnt ligands, GSK3 kinase activity is inhibited resulting in increased levels of cytoplasmic and nuclear β-catenin.

Activation of the pathway can also be caused by inactivating mutations in genes encoding proteins needed for normal targeting of β-catenin for degradation or activating mutations in the β-catenin gene itself.(5) These increased levels of β-catenin form complexes with members of the LEF/TCF family of DNA-binding proteins and activate promoters of numerous target genes.(21) Recently, two novel signaling molecules were linked to regulation of canonical Wnt signaling. Activation of canonical Wnt signaling through Lrp6 requires phosphorylation of Lrp6 on serine residues within the cytoplasmic domain. Pan et al.(22) have reported that Wnt3a induces activation of two lipid kinases that generate the signaling molecule phosphoinositol 4,5-bisphosphate, which in turn regulates phosphorylation of Ser^1490^ on Lrp6. Wu et al.(23) have reported that the small GTPase Rac1 is needed for phosphorylation of β-catenin on serines 191 and 605 through JNK2 (c-*jun* N-terminal protein kinase 2), a step required for nuclear localization of β-catenin.

In addition to canonical signaling, several Wnt proteins activate noncanonical pathways that do not target β-catenin.(24) Different signaling molecules are reportedly engaged by different Wnts in bone cells through the noncanonical pathway. Wnt5a has been reported to activate a histone lysine methyltransferase, STEDB1, through a Nemo-like kinase that is downstream from calcium/calmodulin-dependent kinase II. Activation of SETDB1 inhibits peroxisome proliferator-activated receptor (PPAR)γ signaling and stimulates osteoblastogenesis.(25) Wnt3a and Wnt7a have been reported to activate a signaling complex that includes the G-protein Gα_q/11_ and targets the novel protein kinase C family member, PKCδ, which drives osteoblast differentiation.(26)

The signaling functions of Lrp5 and Lrp6 are tightly regulated by a large number of extracellular proteins including members of the Dickkopf (Dkk) family. Dkks are encoded by a four-member gene family in vertebrates, of which three members (Dkk1, 2, and 4) are reported to interact with Lrp5 and/or Lrp6.(27) The proteins are primarily composed of two cysteine-rich domains that are homologous to colipase folds. They bind to regions within the β-propeller motifs of Lrp5 and Lrp6 and inhibit the ability of Wnt ligands to bind and activate downstream signaling. Dkk-based inhibition may occur by directly blocking the ability of Wnt ligands to bind Lrp5 and/or Lrp6, or it may decrease the availability of Lrp5 and Lrp6 at the plasma membrane surface.(27) This latter mechanism is related to the ability of Dkks to simultaneously bind Lrps and members of the Kremen protein family. The formation of heterotrimeric or higher order complexes containing Dkks, Lrps, and Kremens leads to the internalization of the complex and a decrease in the levels of Lrps at the cell surface. This process, in turn, may be regulated by members of the R-spondin family, which may bind directly to the Lrps or to Kremen proteins, inhibiting their ability to interact with Dkks.(28) However, the role of Kremen is still being debated,(29–31) because some studies have suggested that Kremens are not required to mediate the actions of Dkks.

Several other proteins such as sclerostin, R-spondins, and members of the cysteine-knot-type proteins (including connective tissue growth factor and Wise) bind and regulate the activity of Lrp5 and Lrp6.(32) Sclerostin is of particular interest because the gene encoding this protein was originally identified as being mutated in sclerostosis,(33) a human disease characterized by high bone mass and an unusually square jaw,(34) the latter reminiscent of the jaw phenotype observed in some patients bearing the Lrp5_G171V_ gain-of-function mutation (see below). Cranial nerve root entrapment leading to loss of hearing and vision, as well as trigeminal neuralgia, can also occur in this disease. The expression of sclerostin is restricted to osteocytes, making it an appealing therapeutic target for drug therapy of low bone mass (see below). As an example, overexpression of a constitutively active PTH receptor in osteocytes results in inhibition of sclerostin and a striking increase in bone mass, consistent with the hypothesis that some of the anabolic actions of PTH may be mediated by suppressing sclerostin expression.(35) Finally, Lrp-based signaling may also be controlled by regulating the transit of Lrp5 and Lrp6 to the cell surface. This is based on work showing that the Mesoderm specification (Mesd) protein acts as a molecular chaperone and is required for such transit.(36)

## Wnt RECEPTOR COMPLEX AND ITS ASSOCIATION WITH SKELETAL DEVELOPMENT AND REMODELING

Osteoporosis pseudoglioma (OPPG) is a rare syndrome associated with a dramatic reduction in skeletal mass, evident in infancy, that results in devastating fragility fractures. Limited histologic data indicate that bone formation rates are reduced in this disease. OPPG is accompanied by progressive blindness also beginning in childhood. In 2001, inactivating mutations in LRP5 were identified as the causative genetic basis for OPPG.(37) Analysis showed that Lrp5 was expressed by cells in the osteoblast lineage, but not by osteoclasts, and much of the subsequent work has focused on this cell type as the central mediator of its actions on bone. However, as discussed below, the importance of Lrp5-mediated signaling within osteoblasts will have to be reconsidered in light of new evidence.(38) The progressive blindness in these patients is related to a failure of the hyaloid vessels of the lens to regress after birth.(39) Interestingly, other diseases associated with abnormal angiogenesis or vascularization of the eye are also associated with mutations in Lrp5 or Lrp6 or in proteins that interact with the two receptors, including familial exudative vitreoretinopathy,(40–42) Norrie disease,(43) and macular degeneration.(44)

Additional support for the role of LRP5 in bone growth was provided when two groups independently reported that a point mutation in LRP5 (G171V) was present in affected individuals of families showing an autosomally dominant high bone mass trait.(45,46) These two genetically independent families have BMD ∼5 SD above that of unaffected family members and the general population. One of these original two families had a notable skeletal phenotype with straightening of the angle of the jaw, torus palatines, and torus mandibularis. Torus palatinus and torus mandibularis have subsequently been found to be associated with higher bone mass in postmenopausal women.(47) Individuals with the LRP5_G171V_ mutations have normal lifespans, rarely fracture, and have little skeletal morbidity, although one of us has cared for a patient who developed thoracic compression myelopathy caused by bony overgrowth of his vertebral body (K Insogna, unpublished data).

The glycine normally at position 171 in the Lrp5 protein lies within the fourth blade of the first six-bladed β-propeller. The G171V mutation does not directly increase signaling activity; instead, it inhibits the ability of Dkk1, Sost, and potentially other proteins to bind to Lrp5 and inhibit Wnt signaling. In addition, it may interfere with Mesd binding and the transit of Lrp5 to the cell surface, facilitating increased autocrine signaling within the endoplasmic reticulum. Subsequently, at least six other mutations in human *Lrp5* have been identified and correlated with changes in bone mass.(48)

Mutations in *LRP6* have also been linked to changes in bone mass in humans. Members of a family in which a putative partial loss-of-function mutation in *LRP6* was identified were predisposed to early cardiovascular-related death associated with dramatically elevated levels of plasma LDL and triglycerides, hypertension, diabetes, and osteoporosis.(15) In addition, several mutant mouse models with induced or spontaneous mutations in *Lrp6* have been identified and characterized, with some of these mice reported to have alterations in bone development.(49–52)

Given the dramatic skeletal phenotypes of patients bearing mutations in *LRP5* and *LRP6*, there has been considerable interest in determining whether polymorphisms in *LRP5* or *LRP6* contribute to heritability of bone mass. Several small studies have variably reported associations (or no associations) of LRP5 polymorphisms with bone mass at selected skeletal sites in adults and children. However, two large, recent, genome-wide association studies have firmly established an association between allelic variants in *LRP5* and both bone mass and fracture risk.(53,54) Both studies reported a significant association of the SNP rs3736228 (Ala1330Val) with reduced bone mass and an increased risk of fracture.

In addition to the role of Lrp5/6 in inherited syndromes of high and low bone mass, evidence is emerging for a role for these molecules in acquired skeletal diseases. One striking example is myeloma bone disease, which is characterized by increased bone resorption but suppressed bone formation. Tian et al.(55) have reported that the inhibitor of Lrp5 signaling, Dkk1, is secreted by myeloma cells and have provided evidence to indicate that this is the mediator of myeloma-induced suppression of osteoblast activity.

As described below, animal models have provided considerable insight into the mechanisms by which gain of function in Lrp5/Lrp6 signaling results in a high bone mass phenotype. However, in vivo studies in humans examining the mechanisms of action of Lrp5/6 are largely lacking. Qiu et al.(56) have recently reported increased trabecular bone mass in patients bearing the Lrp5_G171V_ mutation, although formal static and dynamic histomorphometry was not reported.

## MOUSE MODELS WITH TARGETED MUTATIONS IN LRP5, LRP6, AND OTHER COMPONENTS OF THE WNT SIGNALING PATHWAY

*Lrp5*^*–/–*^ mice have lower-than-normal BMD and a persistence of the embryonic eye vasculature, accurately modeling human OPPG.(57) Detailed studies on these mice showed reductions in osteoblast proliferation and function, and they also suggested defects at multiple stages of differentiation. This is consistent with reports showing roles for Lrp5 in both terminal differentiation and in the regulation of proliferation of pre- and early osteoblasts. A mouse strain designed to model the high bone mass phenotypes in humans carrying the G171V mutation has been developed using a rat type I collagen promoter to preferentially drive expression of a Lrp5_G171V_ cDNA in osteoblasts. The resulting mice show increased bone mass, decreased osteoblast apoptosis, and increased bone strength.(58) Whereas this work clearly showed that this point mutation could increase bone mass when specifically expressed in osteoblasts, its tissue-specific expression did not allow a thorough evaluation of the potential side effects of expressing the mutated protein under its endogenous promoter. Despite carrying a mutation capable of increasing the levels of canonical Wnt signaling, human patients carrying this mutated version of *LRP5* have not been reported to have increased susceptibility to tumorigenesis. However, there are a relatively small number of these patients. Given the current interest in developing agents that pharmacologically mimic such mutations that build bone mass, a detailed study of all tissues in mice carrying this mutation in the endogenous chromosomal location would contribute valuable insights.

Mice carrying a heterozygous null mutation in Lrp6 have low bone mass.(50) Further support for a key role for Lrp6 in regulating bone development has been provided by the analysis of bone defects in mice homozygous for a hypomorphic allele of *Lrp6* (*ringelschwanz*) caused by an R886W point mutation.(51) Mice homozygous for this allele survive to adulthood but show evidence of delayed ossification and osteoporosis. Homozygosity for a null allele of *Lrp6* causes neonatal death associated with a loss of distal limb structures and truncation of the axial skeleton.(59) Further support for the importance of Lrp6 in bone development comes from observations that mutations in both Lrp5 and Lrp6 cause synergistic defects in limb development and bone mass.(50,60)

As mentioned, Lrp5/6 serve as “co-receptors” for the Frizzled family of Wnt receptors. Whereas mutations in Frizzled genes have not been associated with changes in human bone disease, mice carrying genetic deletion of Frizzled-9 have a low bone mass phenotype.(61)

Several laboratories have examined the roles of the downstream components of the Wnt signaling pathway in skeletal metabolism. Specifically, the *β-catenin* and *Apc* genes have been conditionally deleted at different stages of osteoblast differentiation. Deletion of *β-catenin* in differentiated osteoblasts (either through *Osteocalcin-cre*(62) or *Collagen1α1-cre*(63) caused severe osteopenia associated with increased osteoclastogenesis, which correlated with altered expression of RANKL and osteoprotegerin (OPG). In contrast, activation of β-catenin in osteoblasts (either through induction of an oncogenic version of β-catenin(63) or by inactivation of the *Apc* tumor suppressor gene(62) leads to dramatic increases in osteoid deposition and severely impaired osteoclastogenesis. Interestingly, these effects on osteoclasts are not seen in humans or mice lacking *LRP5*.(37,57) Mice with deletions of β-catenin in mesenchymal condensations have also been created by either *Dermo1-cre* or *Prx1-cre*–mediated gene deletion. In this context, loss of β*-catenin* leads to an arrest of osteoblast differentiation and enhanced chondrogenesis,(64–66) implying that β-catenin is needed for the initial specification of osteoblasts.

Complementing the overexpression and gene deletion studies in which agonists of the Wnt signaling cascade have been manipulated, are studies in which endogenous inhibitors of Wnt signaling have been altered. Consistent with the notion that Dkk1 plays an important role in suppressing Wnt signaling, haploinsufficiency for Dkk1 results in a high bone mass phenotype in mice.(67) Surprisingly, the Dkk2 knockout mouse does not evidence alterations in bone mass but rather has a mineralization defect, suggesting an unexpected contribution of this molecule to osteoblasts differentiation.(68) Targeted overexpression of another inhibitor of Wnt signaling, secreted frizzled related protein-4 (SFRP4) in osteoblasts, results in a low bone mass phenotype.(69) Transgenic overexpression of the Wnt antagonist Kremen 2 in osteoblasts also severely impairs bone formation.(70) Finally, deletion of the secreted Wnt signaling inhibitor, Sfrp1, leads to the development of high bone mass in older mice.(71)

In addition, emerging evidence suggests an important role for Lrp5 in modulating the response to mechanical strain in vivo. Mechanotransduction is thought to be mediated principally by osteocytes, and work of Sawakami et al.(72) has shown that the anabolic response to mechanical strain in normal mice was completely lost in mice in which the *Lrp5* gene had been deleted. The link between mechanotransduction and Wnt signaling may in part be dependent on sclerostin, the endogenous Wnt-signaling inhibitor, which is expressed in osteocytes. Osteocyte expression of sclerostin is suppressed by mechanical strain.(73)

As noted above, studies examining how Lrp5/6 signaling affects bone metabolism have been entirely predicated on the assumption that altered Wnt signaling in bone cells is the basis for the observed phenotypes. However, Lrp5 is widely expressed,(3) and the Wnt proteins have wide tissue distributions as well. Recently, Yadov et al.(38) reported dramatic findings that challenge the “osteoblast-centric” model of Lrp5 action. As noted, deletion of *Lrp5* in mice leads to a low bone mass phenotype. Yadav et al. found that targeted deletion of Lrp5 in the duodenal enterochromaffin cells of mice results in high circulating levels of serotonin caused by upregulation of the rate-limiting enzyme for serotonin synthesis, tryptophan hydroxylase (Tph1). Dietary restriction of tryptophan resulted in reduced circulating levels of serotonin in *Lrp5*-deficient mice and normalized the suppressed bone formation parameters in these animals, suggesting that the increase in serotonin production might be responsible for this effect. Consistent with this idea, restricted expression of Lrp5_G171V_ in the duodenum resulted in a high bone mass phenotype. These investigators went on to show that the serotonin receptor Htr1b mediates the actions of serotonin in osteoblasts, because selective deletion of this receptor in osteoblasts resulted in a high bone mass phenotype. Interestingly, using a genetic approach they provide evidence that the mechanism by which Lrp5 regulates Tph1 activity in the duodenum is not through a β-catenin pathway. These data suggest that a new endocrine loop, from the gut to bone in which serotonin is the effector hormone, may play a central and hitherto unappreciated role on bone metabolism. They also call into question the role of Lrp5 in bone cells. Recent clinical evidence has suggested an important role for the serotonergic system in regulating bone mass, with reports of reduced bone mass and an increased risk of fracture in patients taking selective serotonin reuptake inhibitors (SSRIs).(74–76) SSRIs are not thought to alter serum serotonin levels, so the relationship of this clinical observation to the findings of Yadav et al. remains unclear.

## THERAPEUTIC MANIPULATION OF THE Wnt Lrp5/6 SIGNALING CASCADE

Because gain-of-function mutations in *LRP5* result in a high bone mass phenotype in which the quality of the bone is good and fracture risk is very low, there is considerable interest in attempting to therapeutically manipulate Wnt signaling in patients with low bone mass. Initial efforts have focused on suppressing endogenous inhibitors of the Wnt signaling system.

Sclerostin has emerged as an attractive therapeutic target because its expression is restricted to osteocytes. A neutralizing antibody to sclerostin has shown promise in initial preclinical and early clinical trials.(77,78) Experimental animals showed an increase in bone formation rates and bone mass during treatment with this antibody, and postmenopausal women treated with a neutralizing antibody showed an increase in markers of bone formation. A neutralizing antibody to Dkk1 has also shown promise in a preclinical trial.(79) A small-molecule inhibitor of SFRP1 has been shown to stimulate bone formation in mice.(80) Thus, drugs targeting these endogenous inhibitors of Wnt signaling may be the first useful therapies to emerge from our new appreciation of the importance of this signaling cascade in bone.

Because PTH is the only available anabolic agent for bone, the relationship between its use for that purpose and Wnt-mediated bone anabolism has been an area of considerable investigative interest. A number of studies suggest that these two signaling pathways do indeed overlap. Recent examples include preliminary reports that PTH is able to stimulate a β-catenin**–**responsive reporter construct in vitro and that PTH induces interaction of the PTH receptor, PTHR1, with LRP6.(81) The formation of a ternary complex of ligand, receptor and Lrp6, is associated with phosphorylation of Lrp6. In terms of building and maintaining bone, the relative contributions of PTH from activating Lrp6 and from downregulating the expression of sclerostin(35,82) will undoubtedly be the subject of intense future study.

**FIG. 1:**
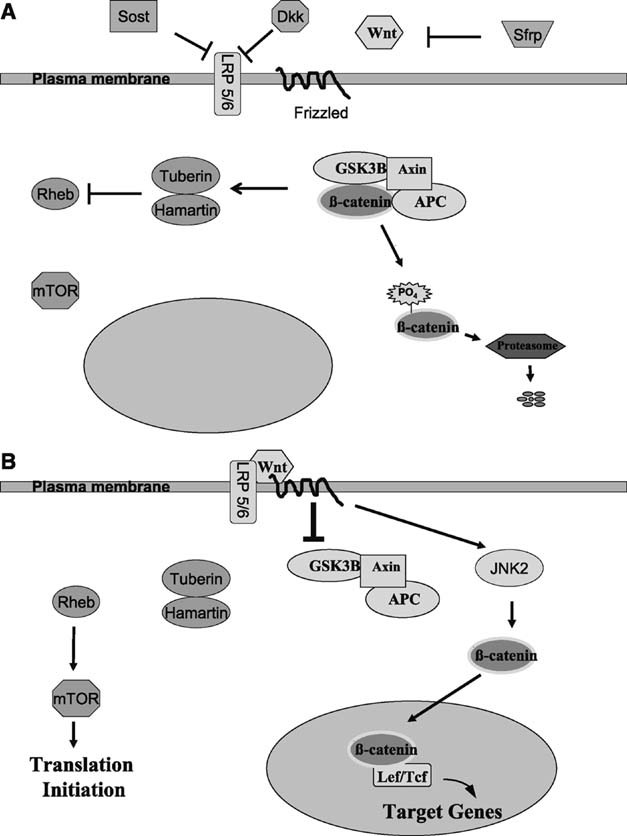
The canonical Wnt (Wnt/β-catenin) signaling pathway (adapted from Ref. 84). (A) In the absence of an upstream inhibitory signal induced by Wnt ligands, the constitutively active serine/threonine protein kinase, glycogen synthase kinase 3 (GSK3α or GSK3β), phosphorylates β-catenin on conserved serine or threonine residues near its amino terminus. This process depends on the formation of a large, multiprotein complex that includes the Axin protein and the product of the *Adenomatous polyposis coli* (*APC*) gene. Phosphorylation of β-catenin targets it for degradation through a ubiquitin-dependent proteolytic pathway. In addition, GSK3 activity also controls the activity of the tuberous sclerosis complex, consisting of the tuberin and harmartin proteins (encoded by the *TSC1* and *TSC2* genes, respectively). GSK3-mediated phosphorylation of TSC1 inhibits this complex, leading to downregulation of the small GTPase, Rheb. This, in turn, results in decreased activity of the mTOR complex (which is activated by Rheb-GTP). Because mTOR normally activates proteins that increase the cellular capacity for translational activity, the net effect of GSK3 activity in the context of canonical Wnt signaling is to downregulate both transcriptional (through β-catenin target genes) and translational (through mTOR regulation) activity. (B) In the presence of an upstream Wnt ligand, GSK3 activity is inhibited, leading to increased cytoplasmic levels of β-catenin, which enters the nucleus and activate transcription of target genes. In addition, inhibition of GSK3 also leads to activation of the mTOR pathway. A parallel pathway leads to the JNK2-dependent phosphorylation of β-catenin, which facilitates nuclear localization. Thus, the net result of activation of canonical Wnt signaling is to coordinately increase both transcriptional (through increased activation of β-catenin target genes) and translational (through activation of mTOR) activation within cells. Activation of the pathway is normally constrained by the expression of proteins such as members of the Dickkopf family and sclerostin, which selectively bind to either Lrp5 or Lrp6, thereby blocking the ability of Wnt ligands to interact with these receptors. In addition, proteins of the secreted frizzled related protein (Sfrp) family can directly bind to Wnt ligands, offering another way to inhibit activation of the pathway.

## CONCLUSIONS

The past 7 yr has seen an explosion in our understanding of the mechanisms underlying the role of Wnt signaling in the regulation of bone mass. The recent work of Yadav et al. that indicates a central role for circulating serotonin in regulating bone formation suggests that inhibiting the actions of serotonin in bone or inhibiting its duodenal synthesis may be new therapeutic targets for treating low bone mass conditions. These observations will undoubtedly stimulate a flurry of activity directed at whether and how the effects of serotonin and the documented role of Wnt/β-catenin signaling within osteoblasts interact to control bone development and homeostasis.

Finally, Lee et al.(83) have also reported that the skeletal system can play a direct role in regulating metabolism through the actions of osteocalcin on pancreatic islets. Coupled with reports that mutations in Lrp5 or Lrp6 in humans and in mice impair the ability to normally regulate serum levels of lipoproteins and glucose,(13–15) it is tempting to speculate that there might be some connection between the role of Lrp5 and Lrp6 in regulating skeletal development and the role of the skeleton as an endocrine organ. In any case, it is likely that the role of Wnt signaling in regulating the skeleton will be a large focus of ongoing research efforts and that major surprises are in store as we continue to explore this complex network.
